# Atypical thyroid tests in an athlete treated for hypothyroidism as the first symptom of pituitary dysfunction due to relative energy deficiency

**DOI:** 10.1530/EDM-24-0066

**Published:** 2024-10-29

**Authors:** Monika Skrzypiec-Spring, Justyna Kuliczkowska-Płaksej, Adam Szeląg, Marek Bolanowski

**Affiliations:** 1 Department of Pharmacology, Wroclaw Medical University, Wrocław, Poland; 2 Department of Endocrinology, Diabetes and Isotope Therapy, Wroclaw Medical University, Wrocław, Poland

**Keywords:** relative energy deficiency, Thyroid, hypogonadism

## Abstract

**Summary:**

Relative energy deficiency in sport occurs in athletes who have limited energy availability. Its typical features include reversible suppression of the hypothalamic–pituitary–gonadal axis. In addition, it may be accompanied by hepatic resistance to growth hormone, leading to a decrease in insulin-like growth factor 1 and dysregulation of the hypothalamic–pituitary–thyroid axis. We present the clinical case of a 33-year-old athlete previously treated effectively for hypothyroidism, who presented with low thyroid-stimulating hormone, low free triiodothyronine, and normal free thyroxine. Based on diet and training interviews and further laboratory tests, dysregulation of the hypothalamic–pituitary–thyroid axis and reversible hypogonadism due to insufficiency of energy available to support energy expenditure were revealed. We also discuss here challenging diagnostic dilemmas that may appear in athletes of normal body weight but result from insufficient energy supply in relation to demand, and review the literature for the clinical course and possible mechanisms underlying the relative energy deficiency.

**Learning points:**

- Atypical thyroid function tests in athletes may be the first manifestation of reversible pituitary dysfunction due to relative energy deficiency.
- Typical symptoms of relative energy deficiency include reversible suppression of the hypothalamic–pituitary–thyroidal axis and hypothalamic–pituitary–gonadal axis.
- Relative energy deficiency may occur in all people who train intensively, regardless of their body weight and BMI, as well as in people who have rapidly lost weight as a result of a low-calorie diet and intense exercise.

## Background

Negative caloric balance and low body weight can impair the function of the pituitary gland. This syndrome, previously recognized in female athletes and referred to as the triad of female athletes, is currently also diagnosed in males, and since 2014, according to the position of the International Olympic Committee, is referred to as relative energy deficiency in sport (REDS) (1). A typical symptom of REDS is reversible suppression of the hypothalamic–pituitary–gonadal (HPG) axis and the hypothalamic–pituitary–thyroidal (HPT) axis. In addition, it may be associated with several additional endocrine changes, including hepatic growth hormone resistance leading to decreased IGF-1 and dysregulation of thyrotropic feedback control (2).

REDS is a difficult diagnostic problem for doctors who do not work with athletes on a daily basis. It may occur in both professional athletes and amateurs who train intensively, even with normal body weight and body mass index (BMI), as well as in people who quickly lose weight as a result of a low-calorie diet and intense physical exercise.

Below, we present the clinical case of a 33-year-old athlete previously treated effectively for hypothyroidism who presented with low thyroid-stimulating hormone (TSH), low free triiodothyronine (FT3), and normal free thyroxine (FT4) as a first manifestation of dysregulation of the HPT axis due to energy availability insufficient to support energy expenditure.

## Case presentation

A 33-year-old athlete who had hypothyroidism since the age of 17, otherwise with no significant medical or family history, presented to an endocrinologist and sports medicine specialist because of low testosterone levels and difficulty finding the correct dose of levothyroxine for a year.

From May to October 2022, the patient was on a low-carbohydrate, calorie-restricted diet (calorie intake was approximately 25 000 kcal/week) while simultaneously undergoing four intense strength training sessions of 1 h each and six moderate-intensity aerobic training sessions (running) lasting 40–45 min. He denied using any androgenic-anabolic steroids.

**Figure 1:**
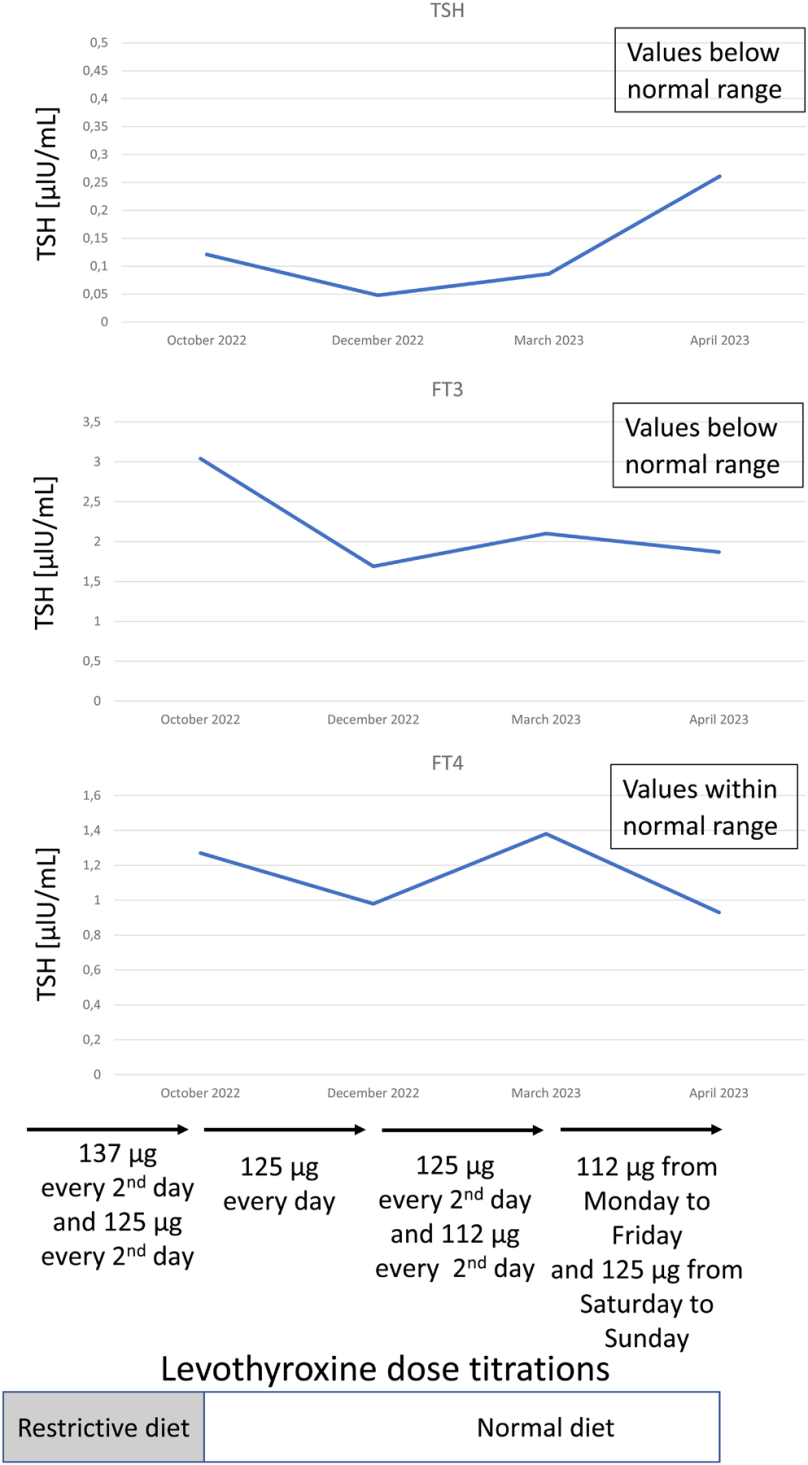
The results of thyroid function tests performed from the time of completing the low-carbohydrate and calorie-restricted diet to April 2023, with changes over time relative to dose titrations. TSH normal values: 0.270–4.200 μlU/mL, FT3 normal values: 2.0–4.4 pg/mL, FT4 normal values: 0.932–1.700 ng/mL.

The patient reported to an endocrinologist and sports medicine specialist for the first time in April 2023 after several unsuccessful attempts to titrate the dose of levothyroxine to address unusual thyroid function results made by other endocrinologists. The results of thyroid function tests performed from the time of completing the low-carbohydrate and calorie-restricted diet to April 2023, with changes over time relative to dose titrations, are shown in Fig. 1.

Briefly, the results obtained in October 2022 (after the end of the low-carbohydrate and calorie-restricted diet) showed a low TSH of 0.121 μlU/mL (0.270–4.200), low FT3 of 3.04 pmol/L (3.100–6.800), normal FT4 of 1.27 ng/dL (0.932–1.700), and normal anti-thyroid peroxidase antibody levels. In a peripheral blood smear, glucose and creatinine levels were normal. The patient underwent an endocrinological consultation. It was then decided to reduce the dose of levothyroxine from 137 μg taken every second day and 125 μg taken every second day to 125 μg taken every day. Control test results performed after 3 months showed a further reduction in the concentrations of TSH and FT3 (0.0478 μlU/mL (0.270–4.200) and 1.69 pg/mL (2.0–4.4), respectively), normal FT4 of 0.98 ng/dL (0.932–1.700), and normal thyroglobulin antibodies. Despite the declining concentrations of free thyroid hormones, the dose of levothyroxine was then reduced, and the patient was ordered to take 125 μg every second day and 112 μg every second day. Controlled results performed in March 2023 showed low TSH of 0.086 μlU/mL (0.270–4.200), normal FT3 of 3.24 pmol/L (3.1–6.8), and normal FT4 of 17.8 pmol/L (11.9–21.6). The levothyroxine dose was reduced, and the patient was ordered to take 112 μg/day from Monday to Friday and 125 μg/day on Saturday and Sunday.

When the patient reported to an endocrinologist and sports medicine specialist in April 2023, his body weight was 3 kg higher than it was in October 2022, but his workouts had not changed. The physical examination revealed an athletic build, height of 175 cm, weight of 75 kg, and BMI of 24.49. The results of the tests performed at that time showed low TSH of 0.261 μlU/mL (0.270–4.200), normal FT3 of 1.87 pg/mL (2.0–4.4), normal FT4 of 0.93 ng/dL (0.932–1.700), low total testosterone of 2.26 ng/mL (2.40–8.71), normal luteinizing hormone (LH) of 2.25 mlU/mL (0.57–12.07), normal follicle-stimulating hormone (FSH) of 1.9 mlU/mL (0.95–11.95), normal estradiol of 67.9 pmol/L (41.4–159), and normal prolactin of 116 mlU/L (86–324). Thyroid ultrasound and semen analysis performed in March 2023 were within normal limits.

## Investigation

The patient was suspected of reversible pituitary gland dysfunction resulting from relative energy deficiency. In order to fully assess the pituitary function, the patient was referred for cortisol, ACTH, and IGF-1 assessments and magnetic resonance imaging of the pituitary gland. Also, the patient was recommended to perform anti-TSH receptor antibodies (TSHrAb) tests. The results showed: normal cortisol of 19.8 μg/dL, normal ACTH of 15.6 pg/mL (7.2–63.3), normal IGF-1 of 202 ng/mL (71–234), and normal TSHrAb <0.80 IU/L (<1.50). MRI of the pituitary gland was normal. The patient was advised to follow a diet with a positive calorie balance and to take levothyroxine 100 μg taken every second day and 112 μg taken every second day.

The response of LH, FSH, and total testosterone to 50 mg of clomiphene daily administered for 10 days was evaluated, showing an increase of LH to 4.35 mIU/mL (1.00–95.60), FSH to 2.51 mIU/mL (1.70–23.00), and total testosterone to 4.710 ng/mL (2.180–9.060). At this point, a diagnosis of reversible pituitary gland dysfunction resulting from relative energy deficiency was confirmed.

## Treatment

The patient was recommended to continue levothyroxine 100 μg taken every second day and 112 μg taken every second day and to take 25 mg of clomiphene every second day, and perform the controlled laboratory tests in 1 month.

## Outcome and follow-up

The controlled results performed after 1 month revealed: normal TSH 0.89 mIU/mL (0.27–4.2), low FT3 of 1.84 pg/mL (2.0–4.4), normal FT4 of 1.07 ng/dL (1.0–1.6), normal LH of 4.94 mIU/mL (1.7–8.6), normal FSH of 2.84 mIU/mL (1.5–12.4), and normal testosterone of 4.64 ng/mL (2.49–8.36). The therapy was continued for the next 3 months.

## Discussion

REDS is a syndrome whose diagnosis can cause problems for doctors who do not work with athletes on a daily basis. It results from an incorrect supply of energy relative to its consumption. Historically, the clinical symptoms in sportswomen resulting from insufficient energy supply compared to energy expenditure were known as the triad of sportswomen. Typical symptoms of this syndrome included menstrual disorders, eating disorders, and decreased bone mineral density due to impairment of the HPG axis. The probable mechanisms responsible for pituitary dysfunction include the inhibition of hypothalamic secretion of gonadotropin-releasing hormone (GnRH) due to a decrease in serum leptin concentration related to the reduction of fat mass or acute restriction of energy intake (2, 3, 4, 5). Moreover, it is postulated that leptin at least partially acts via kisspeptin by reducing kisspeptin-induced activation of the HPT axis. Other suggested mechanisms include the influence of elevated ghrelin concentration, activation of the hypothalamic–pituitary–adrenal axis, and a proinflammatory state (2).

The underlying etiology of low energy availability is the same in men, but probably due to a more subtle presentation for a long time, it was not recognized in men. REDS may occur in people with normal body weight and BMI (2). That is what happened in our case. When HPT axis regulation disorders in the form of low TSH with a low concentration of free hormones were found, the patient had normal weight and BMI. Because of the gender, other components of this syndrome, such as a disorder of the HPG axis, did not have a clear clinical manifestation (in women, the suspicion of this syndrome is facilitated by the presence of menstrual disorders). This may explain why the consulting endocrinologist did not suspect REDS and did not order laboratory tests to evaluate this axis. Due to the lack of a correct diagnosis, he also did not start the proper treatment and instead decreased the dose of levothyroxine, despite clearly too low concentrations of free thyroid hormones, probably guided only by low TSH concentrations. This resulted in unsuccessful attempts to determine the dose of levothyroxine lasting several months. The assessment of the patient's HPG axis function was performed independently as part of the training-related laboratory control, as there were no clear clinical signs of functional hypogonadism. Completion of the diet with a low supply of calories and carbohydrates resulted in an increase in body weight, equalization of energy supply and demand, and contributed to the improvement of the HPT axis because, at the time of appearance at the sports medicine specialist and endocrinologist, both TSH and free hormones remained within standard limits. Low testosterone levels with inadequate gonadotropin levels indicated a disorder in the secretion of gonadotropins by the pituitary gland. The patient was suspected of functional pituitary dysfunction as a result of REDS. It was recommended to evaluate other pituitary hormones and IGF-1 to assess the response of the liver to growth hormone.

In the case of functional hypogonadism caused by REDS, non-pharmacological treatment is recommended, consisting of dietary changes. In the clinical cases described so far, such treatment leads to the normalization of testosterone concentrations within a few months of starting this treatment (6, 7). Nevertheless, in the described patient, despite dietary management for 6 months and an increase in body weight by 3 kg, low testosterone concentrations, and inadequately low LH and FSH concentrations persisted. Due to this, despite the justified suspicion that this patient's hypogonadism was functional, a clomiphene stimulation test was performed in order to differentiate the diagnosis from the non-functional nature of hypogonadotropic hypogonadism, as well as to assess the possible success of treatment. Clomiphene exerts its action in the hypothalamus as a competitive estrogen receptor modulator. Estradiol is responsible for part of a negative feedback mechanism which inhibits the production and release of GnRH. Clomiphene competes with estradiol at the hypothalamic and hypophyseal receptor levels and it increases GnRH release. This stimulates the anterior pituitary to release LH and FSH. The clomiphene stimulation test was validated for functional hypogonadotropic hypogonadism by Rabijewski, who showed that in men with functional hypogonadotropic hypogonadism, increases in testosterone levels in the clomiphene test >150%, LH >100%, and FSH >50% were associated with increased testosterone levels if treatment with clomiphene at a dose of 50 mg daily was continued for 3 months (8). The effectiveness of clomiphene in the treatment of functional hypogonadotropic hypogonadism has been confirmed in numerous studies, although this is not standard therapy (9, 10, 11, 12, 13, 14). The increase in the concentration of LH and FSH and testosterone in the described patient confirmed the functional nature of hypogonadism and the possibility of a good response to treatment. Additionally, MRI was ordered to rule out organic causes of pituitary dysfunction. Due to the persistence of hypogonadism after 6 months of non-pharmacological treatment and weight gain, a decision was made to continue clomiphene treatment with gradually reduced doses, although it is still not approved therapy and it could be considered only in specific situations.

In conclusion, REDS is an underestimated clinical problem that affects not only athletes but also patients who do not practice sports, due to a disturbed proportion of energy expenditure to its supply, which is becoming more and more common in the context of body worship and the resulting dietary restrictions. Because it may significantly disrupt endocrine function, an indispensable element of the endocrine interview should include questions about nutrition and physical activity, especially when the patient has atypical laboratory test results. Endocrinological tests should not be performed on patients with energy deficiency in relation to energy expenditure, as the results cannot be clearly interpreted and need to be reassessed once normal energy balance has been restored.

## Declaration of interest

The authors declare that there is no conflict of interest that could be perceived as prejudicing the impartiality of the study reported.

## Funding

This work did not receive any specific grant from any funding agency in the public, commercial, or not-for-profit sector.

## Patient consent

Written informed consent for publication of their clinical details and/or clinical images was obtained from the patient.

## Author contribution statement

MS-S: collecting data, writing the paper; JK-P: writing the paper; AS: critical revision; MB: critical revision, supervising.
